# Knocking on heaven's door: The gap between health institutions and academies in generating knowledge utilizing real-world data

**DOI:** 10.3389/fpubh.2022.1002910

**Published:** 2022-10-26

**Authors:** Giovanni Corrao, Matteo Franchi, Giuseppe Mancia

**Affiliations:** ^1^National Centre for Healthcare Research and Pharmacoepidemiology, Department of Statistics and Quantitative Methods, University of Milano-Bicocca, Milan, Italy; ^2^Unit of Biostatistics, Epidemiology and Public Health, Department of Statistics and Quantitative Methods, University of Milano-Bicocca, Milan, Italy; ^3^Centro Cardiologico Monzino IRCCS, Milan, Italy; ^4^University of Milano-Bicocca, Milan, Italy

**Keywords:** real-world data, real-world research, decision-making, data accessibility, data interconnection

Albeit with an inevitable delay from the eruption of the first wave of the Coronavirus disease 2019 (COVID-19) pandemic, medical research has offered populations and patients monumental achievements that should not be forgotten. The virus has been isolated and its variants can be promptly isolated as they appear. Rapid and increasingly more-reliable diagnostic tests have been made available at progressively lower prices. The diverse local and systemic manifestations of COVID-19 viral infections have been described in detail together with the multiple risk factors responsible for their evolution to severe and lethal forms. Direct (antiviral drugs and monoclonal antibodies) and indirect treatment strategies have been discovered or refined with a measurable benefit on infection-related outcomes. The long-term consequences of COVID-19 remain mechanistically unclear, but their multiple clinical phenotypes have been characterized and are now better understood.

Finally, and most importantly, efficacious and safe vaccines against COVID-19 have been developed at a speed never achieved in the past, thanks to the huge efforts of the pharmaceutical industry backed by government support (1). Well-designed randomized clinical trials have provided robust evidence of the immunogenicity, efficacy, and safety of most available vaccines (2–8), and vaccination campaigns have successfully involved large population strata in numerous countries, despite multiple critical issues and difficulties. Somewhat unexpectedly, vaccination has turned out to have limited ability to prevent variant-related COVID-19 infections, but the documented efficacy of vaccination against severe and lethal diseases has now prevented hospitals from becoming overwhelmed (9–12). In summary, it is undeniable that the world is much better equipped to fight future pandemics.

Despite the above-mentioned contributions, research has not provided unequivocal answers to several important basic as well as diagnostic and therapeutic questions. For example, concerning vaccination, unresolved questions abound. First, how should individuals most prone to severe infection be identified and prioritized for vaccination? (13). Second, what are the reasons for inequality in the distribution of vaccination within populations? (14). Third, to what extent do vaccination programs prevent COVID-19 infections, hospitalizations, or death, and how does this change in relation to emerging variants? (15, 16). Fourth, what harms are associated with COVID-19 vaccines and how can rare, serious adverse effects be explained, predicted, and avoided? (17). Lastly, what is the actual duration of vaccine-dependent protection and how can we measure the various components and overall efficacy of vaccination? (18). These issues have a direct impact on the population's health worldwide.

These outstanding issues justify the efforts to continuously monitor and assess vaccination campaigns beyond the classical pharmacovigilance approach that has been implemented in several countries, including Israel, the United States, the United Kingdom, and Italy (15, 16, 19–21). In some countries, efforts have also been made to do research into COVID-19 by using a coordinated or integrated approach. In these integrated research pathways, basic science helps in the identification of preventive and curative agents, experimental trials are carried out to prove efficacy and safety, and real-world observations complement trial evidence. This approach provides integrated information with which to guide the use of new COVID-19 treatments in medical practices and at the level of public health. This implies the recognition of real-life-based research as a fundamental component of the knowledge obtained by the research community in the COVID-19 pandemic, with a critical role to play in the future.

The above-mentioned integrated research pathway, and the inclusion of real-life data as a necessary (although insufficient) research step, is not a new approach. What has recently happened as a result of the COVID-19 pandemic has only accelerated a process that had begun at least a decade ago to tackle other medical issues. For example, in precision medicine (22), healthcare strategies are personalized and, therefore, patient subgroups who will benefit most from new drugs need to be identified. Precision medicine requires that the population is characterized by a wide range of individual biomolecular, clinical, demographic, and socio-economic features that can only be made available by real-life information. Real-life data is also required for the adoption of treatment guidelines. Even when these guidelines are evidence-based, their recommendations need to be verified in the context of real-life application to measure levels of acceptability and adherence by physicians and patients, as well as the appropriateness of their use (23).

Even today, many medical actions are dictated by tradition or mechanistic inferences, rather than by trial findings or other types of evidence; therefore, real-life data from clinical practice may be necessary to verify the actual clinical impact and cost implications of these actions (24). Service implementation, availability, accessibility, and integration can be optimized in so-called “value-based healthcare”, which maximizes clinical benefits at lower costs (25), and this can only be provided by real-world evidence. As conceptualized by Tanahashi in a pivotal publication (26), we need to measure potential coverage (service availability, accessibility, and acceptability), contact coverage (use of evidence-based healthcare recommendations), and effective coverage (use of healthcare data which translates into health benefits) in people who are stratified according to health needs.

However, two main problems may slow down the use and development of observational studies based on routinely collected data. The first problem is that accessibility to (and interconnection of) the now widely available, real-world big data in a large number of countries is questioned by individuals who place the secrecy of health-related information above any other consideration. Of course, an individual's health status should remain strictly confidential, but tools and techniques capable of protecting this information entered research several years ago. Privacy by design (27) is now an integral part of any study protocol that involves data of this type, and universal application of this rule should become mandatory for any future real-world-based research. This being the case, the ethical principle according to which the privacy of each individual citizen should be protected does not justify a rigid (and regretfully widespread) attitude that prevents access to valuable healthcare data, which can be of fundamental importance for medical research and knowledge, even more during pandemics. In countries such as Sweden, this problem has been avoided by asking citizens to authorize the use of their anonymized healthcare data through the no-reply assent approach (28), which has enabled this country to collect widespread clinical registry data of inestimable research value. Hopefully, other countries will soon follow this example.

The second problem is that even when real-life big data is available, the generation of credible evidence is not guaranteed, even if the data source is of good quality. This problem occurs because if some basic rules are not respected, even good-quality data can give a distorted image of reality. One basic rule, for example, is to avoid “a fishing expedition” (29). A researcher throwing the hook into a very fishy sea of data will almost always catch something. However, the goal of meaningful research is not to look for whatever can be caught in the large sea of data (for example, statistical correlations) but to identify causal links that aid the progression of knowledge and improve health system quality. Another basic rule is to avoid what statisticians call data torture [30], which is the re-analyzation of the same data again and again until results plausible for the original hypothesis of the investigator are obtained. Obviously, this has little to do with scientific research.

In conclusion, the COVID-19 pandemic has made it clear that it is time to stop considering observational research based on widely available big data, as at best “hypothesis generating” and realize that these data represent a final fundamental research step without which knowledge obtained by classical research approaches lose much of their potential value. Anyone who is able to design a study that uses current data and their interconnections to improve knowledge and support decision-making, who has documented skills that guarantee rigorous data analysis, who respects privacy, and who makes the results of the study available to health systems should be enabled to access the data. In other words, observational research by means of big data should be available to anyone who guarantees an ethical approach to respect the rules of privacy protection and follows good research practices.

## Author contributions

GC, MF, and GM were responsible for the design of this work. GC drafted the work and is accountable for all aspects of the work to ensure its accuracy and integrity. All authors gave the final approval of the version to be published.

## Conflict of interest

Author GC received research support from the European Community (EC), the Italian Agency of Drugs (AIFA) and the Italian Ministry for University and Research (MIUR). He took part in a variety of projects that were funded by pharmaceutical companies (i.e., Novartis, GSK, Roche, AMGEN and BMS). He also received honoraria as a member of the advisory board to Roche. The remaining authors declare that the research was conducted in the absence of any commercial or financial relationships that could be construed as a potential conflict of interest.

## Publisher's note

All claims expressed in this article are solely those of the authors and do not necessarily represent those of their affiliated organizations, or those of the publisher, the editors and the reviewers. Any product that may be evaluated in this article, or claim that may be made by its manufacturer, is not guaranteed or endorsed by the publisher.
